# Real-life functioning in women with schizophrenia living in residential facilities: Gender-based comparison

**DOI:** 10.3389/fpsyt.2023.1120141

**Published:** 2023-03-21

**Authors:** Olga Rusakovskaya, Natalia Kharitonova, Larisa Movina, Oleg Papsuev

**Affiliations:** ^1^Department of Forensic Evaluation in Civil Procedure, V. Serbsky National Medical Research Centre for Psychiatry and Narcology, Moscow, Russia; ^2^Faculty of Legal Psychology, Moscow State University of Psychology and Education, Moscow, Russia; ^3^Department of Psychotic Disorders, Moscow Research Institute of Psychiatry – Branch of V. Serbsky National Medical Research Centre for Psychiatry and Narcology, Moscow, Russia; ^4^JSC “European Medical Center”, Moscow, Russia

**Keywords:** schizophrenia, real-life functioning, institutional care, gender, functional outcome

## Abstract

**Introduction:**

Despite many patients with schizophrenia being able to achieve good functional outcomes, the number of patients with poor functional outcome estimates at over 25 percent. One of the wider constructs, reflecting functional outcomes in schizophrenia, is real-life functioning, whose key domains include ability to live relatively autonomously, productive activity and social interaction. Negative symptoms are seen among independent predictors of real-life functioning. As most researchers agree that schizophrenia is a disease with gender differences in terms of both clinical and functional outcomes, the goal of our observational study was to examine real-life functioning of women with schizophrenia, living in residential care facilities, and study the relationship between daily functioning and negative symptoms.

**Methods:**

Using the Standardized Protocol of Clinical Interview and observation for 1 or more weeks, we examined 46 females with schizophrenia, living in psychiatric residential facilities and compared them with 54 males with schizophrenia, living in the same facilities. In a pilot study 21 subjects with schizophrenia (13 females and 8 males), were evaluated by the Russian version of the Brief Negative Symptom Scale (BNSS).

**Results:**

To the results, more females with schizophrenia, compared to males with schizophrenia, remained active and took initiative in their physical care, vocational activities, involvement in cultural events, and maintained friendly relationships with other inmates. We have identified a group of inmates, in which females prevailed, with less pronounced negative symptoms and a higher level of social functioning, who did not need residential social care in the institutions.

**Discussion:**

Limitations of residential social care in the institutions for psychiatric patients are discussed.

## 1. Introduction

Schizophrenia is currently conceptualized as a broad array of neurodevelopmental psychotic conditions remaining among the most socially significant mental disorders and top ten causes of disability worldwide. Recent findings have shown that not only positive and negative syndromes affect social roles and functioning in people with schizophrenia but biased neurocognition and social cognition come on stage to become targets for newer interventions (1, 2). At the same time while being the most common and complex clinical manifestations of schizophrenia, negative symptoms are thought to mediate neurocognition and functional outcomes. Despite 13.5 to 50 percent of patients with schizophrenia being able to achieve good functional outcomes with adequate treatment (3–8), the number of patients with poor functional outcome remains high, estimated at over 25 percent (9, 10). Percentage of patients with formal schizophrenia-related disability status can be as high as 40% (6), with even more patients with schizophrenia being actually disabled. Patients’ disability place a significant burden on their families and other meaningful persons due to negative symptoms severity and insufficiency of psychiatric and social care resources to support these people (11, 12).

In 1985 Warner R. proposed that social environment had a significant impact on the outcomes of psychotic disorders: in other words, circumstances that foster greater social inclusion lead to a range of beneficial consequences for better social functioning. It has been shown that functional outcomes in schizophrenia should be considered in two different but interrelated aspects, clinical and social (13).

Reviewing the concepts of social outcomes in schizophrenia, Priebe S. (2007) mentions the following outcomes: standard of living, quality of life, social functioning, social integration, need for care, and social inclusion (14). During their lifetime, patients with schizophrenia, affected by their psychotic disorder, lose the main domains conducive to social inclusion, i.e., productivity and social integration, which has a negative impact on social outcomes of the disease.

One of the wider constructs, reflecting functional outcomes in schizophrenia, is real-life functioning, whose key domains are: (1) ability to live relatively autonomously (no need in residential social care); (2) productive activity; and (3) social interaction. In all three domains, three components are necessary for successful functioning: certain functional skills, motivation for using these skills, and ability to adequately assess the situation in which using these skills is appropriate (15). In the model proposed by Galderisi S. and co-authors (2014), which, in our opinion, is the most comprehensive and robust, real-life functioning in schizophrenia as a latent variable has independent predictors such as The Positive and Negative Syndrome Scale (PANSS) positive symptoms and disorganization, The Brief Negative Symptom Scale (BNSS) avolition, neurocognition, and incentives; and mediators such as social cognition, functional capacity, internalized stigma, resilience, and service engagement (16).

According to a review by Giordano G. and co-authors (2021), most researchers agree that schizophrenia is a disease with gender differences in terms of both clinical and functional outcomes (17). One possible explanation is the estrogen hypothesis which takes in account the role of sex hormones that can possibly affect the course of schizophrenia in females. Oestrogens are thought to be a protective factor that delays the onset of schizophrenia, allows females to gain better social skills during adolescence and develop less prominent negative and neurocognitive symptoms (18). McGrath J. and co-authors (2004) have shown that women tend to develop schizophrenia less often than men (19). However, compared to men, the onset of schizophrenia in women occurs later than that in men, which determines more favorable premorbid characteristics in the social and academic domains. Negative symptoms are less pronounced in women than in men. The incidence of comorbid mental disorders associated with alcohol and substance abuse is also lower in women (17, 20–22) and life expectancy is higher. Hence, functional outcomes in women with schizophrenia are expected to be better than that in men, however the ‘protective’ impact of estrogen levels for women reduces around the perimenopause which may impact negatively on functional outcomes (18).

Thus, women with schizophrenia living in residential institutions represent a cohort of patients with poor functional outcomes, on the model of which it is possible to study independent predictors and mediators of daily functioning, as well as the influence of oestrogens on this parameter.

The aims of the study were to (i) compare real-life functioning of men and women with schizophrenia living in residential care facilities (ii) describe the relationship between daily functioning and negative symptoms in women with schizophrenia.

## 2. Materials and methods

### 2.1. Participants

The sample included 100 persons, living in residential psycho-neurological care facilities located in three central regions of the Russian Federation.

“Psyco-neurological” care facilities, alike nursing homes, are social institutions, where people suffering from a chronic mental disorder live, due to their inability to live independently. There are three prerequisites for placement in a psycho-neurological care facility: inability to live independently, chronic and severe mental disorder, the conclusion of a psychiatric commission that, due to a mental state, a person cannot live in a facility for the elderly and disabled due to non-mental disorders. In psycho-neurological care facilities, residents are fully provided with food, clothing and other household needs, their life is built into the routine of certain rehabilitation procedures measures. However, in the recent past, the goals of such rehabilitation work did not include the development or restoration of a person’s ability to live independently, and residential psycho-neurological care facilities were considered as institutions in which a person who entered them would live permanently, until his or her death.

The main group comprised 46 females with schizophrenia. The comparison group comprised 54 males with schizophrenia, living in the same facilities. Both male and female subjects with schizophrenia had been placed in residential psycho-neurological care facilities at the age of 18 to 65 because of severity and chronic nature of their symptoms, pronounced social disadaptation, inability to live independently, and lack of sufficient social support from family members. In these subjects, the disease was characterized either by frequent exacerbations of mental disorder along with the absence of insight into symptoms and the lack of treatment compliance, or by severity of negative symptoms that also led to impaired functional capacity. All subjects with schizophrenia were on mental disability pension and 42% of these subjects were deemed legally incapable.

All subjects signed a written informed consent to participate in this study after receiving a comprehensive explanation of the study procedure and goals.

### 2.2. Inclusion and exclusion criteria

Inclusion criteria: Diagnosis of schizophrenia according to International Classification of Diseases, Tenth Revision (ICD-10) criteria; orderly behavior; stable physical condition; the ability to provide informed consent to participate in the study.

Exclusion Criteria: aggressive and/or inappropriate behavior; exacerbation of a somatic disease; inability to live independently or in a supported environment in the community; refusal/inability to provide informed consent to participate in the study.

The sample included persons who, while living in residential psycho-neurological care facilities, were considered by the staff of the institution as “more secure,” and for whom the possibility of a potential transfer to a non-stationary form of social service, if such a form of social service is organized in the region, was not excluded. Thus, the exclusion criterion was the fundamental inability of people with schizophrenia under any conditions to live relatively autonomous.

### 2.3. Study design

This was a naturalistic, observational study that consisted of at least a week of observation, following interview of participants, interview of informants (residential facility staff), data generation, statistical processing and analysis of the results.

All study participants who met inclusion criteria (*n* = 100) were clinically interviewed with the Standardized Protocol and 21 of them to the date of data cut were also assessed by the Russian version of the Brief Negative Symptom Scale (BNSS) by a single researcher (O.R.).

The interview with each subject lasted for at least one hour. The protocol was filled out not only on the basis of the answers of the subjects, but also taking into account personal observation and medical documentation.

During the period of at least one week of observation the researchers were daily in the institution from 9:00 to 18:00, communicating with residents, talking to them on various topics, participating in all routine daily activities. After establishing contact with the subject, we asked to show the room in which she or he lives, to tell how everything was arranged for them.

### 2.4. Assessment scales

Standardized Clinical Interview Protocol, developed by the authors of this study as a tool to bring uniformity to the collection of information about subjects in forensic psychiatric research in connection with the issue of legal capacity and approved by the Local Ethic Committee of the V. Serbsky National Medical Research Centre for Psychiatry and Narcology (23). The Standardized Clinical Interview Protocol consists of 69 items, organized into 7 blocks. The first block (11 items) is intended for collecting general information: personal data; mental health diagnosis; nature of the query (forensic psychiatric evaluation in connection with an application for changing an individual’s capability status; consultation with a psychiatrist, or a psychiatric evaluation to decide on an appropriate form of social care); actual capability status (full legal capacity, diminished legal capacity, or fully legally incapacitated); and previous attempts to change capability status.

The second block (9 items) is concerned with the person’s life and medical history: education, jobs, marital status, alcohol or substance abuse, history of criminal charges, disease onset age, overall number of hospitalizations to inpatient psychiatric wards and institutions, and placement in a residential psychoneurological social care facility (if the subject lives in such an institution).

The third block (19 items) is concerned with the assessment of the person’s actual mental state.

The fourth block (7 items) targets the course of the disease over the last 3 years as well as medication therapies.

The fifth block (5 items) evaluates the level of basic school skills, awareness of important sociopolitical events, and financial awareness.

The sixth block (11 items) evaluates everyday functioning. The items deal with basic, household and instrumental everyday activities, based on the model of activities of daily living (24), preferred leisure activities, inclination toward productive activities (work, hobbies, education, attending sports classes and groups, attending cultural events).

The seventh block (7 items) is concerned with information about the subject’s social contacts and interactions.

Twenty-one subjects with schizophrenia (13 females and 8 males), were evaluated by the Russian version of the Brief Negative Symptom Scale (BNSS) (25, 26). The 13 items of the Brief Negative Symptom Scale are grouped into six subscales: anhedonia (intensity of pleasure during activities/frequency of pleasure during activities/intensity of expected pleasure from future activities), distress, asociality (behavior/internal experience), avolition (behavior/internal experience), blunted affect (facial expression/vocal expression/expressive gestures), alogia (quantity of speech/spontaneous elaboration). For all items on the six subscales, the highest scores correspond to the greatest disturbances. The total score on the scale is calculated by summing the 13 individual items, subscale scores are calculated by summing the individual items within each subscale. The Russian version of the Brief Negative Symptom Scale was validated in a pan-European multicentre study.

### 2.5. Recorded variables

The first and the second blocks of the Standardized Protocol were used to obtain data on demographic and illness-related variables. Data on age, education, work experience, disability, legal incapacity, presence of a family, length of stay in the residential facility, diagnosis, number of hospitalizations, substance abuse were obtained for all 100 patients.

The data on demographics and illness-related variables are provided in Table 1. The group of females with schizophrenia and males with schizophrenia were comparable in terms of age (*t* = −0.56; *p* = 0.58), level of education, duration of living in a residential psycho-neurological care facility (*t* = 0.9; *p* = 0.37).

**Table 1:** The characteristics of the study samples.

	Females with schizophrenia (*N* = 46)	Males with schizophrenia (*N* = 54)
Age (years, mean ± SD)	50.5 ± 13.4	51.9 ± 11.6
Receive disability pension (% yes)	100	100
Legally incapable (% yes)	43.5	38.9
Diminished or limited legal capacity (% yes)	0	1.9
Did not receive any education (% yes)	0	0
Special education according to an adapted program for children with intellectual disabilities (% yes)	0	10.3
Basic education (% yes)	26.9	17.2
Secondary or vocational education (% yes)	30.8	41.4
Higher education (% yes)	42.3	31.0
Have been employed (% yes)	88	72.4
Have been married (% yes)	28.3	24.1
Have children (% yes)	13	3.7
Were held criminally liable (% yes)	2.2	14.8
Were declared insane and were on compulsory treatment (% yes)	0	5.6
Have history of alcohol or drug abuse (% yes)	28.2	29.6
Number of hospitalizations to inpatient psychiatric wards and institutions less than 3 (% yes)	10.8	9.3
Number of hospitalizations to inpatient psychiatric wards and institutions more than 3 and less than 10 (% yes)	21.7	11.1
Number of hospitalisations to inpatient psychiatric wards and institutions more than 10 (% yes)	67.4	79.6
Were transferred to residential psycho-neurological care facilities from children’s homes for children with mental disorders (% yes)	0	1.9
Age of admission to residential psycho-neurological social care facility(years. Mean ± SD)	46.4 ± 13.3	45.4 ± 15.4
Duration of living in a residential psycho-neurological care facility (years. Mean ± SD)	8.5 ± 5.9	7.05 ± 4.9

The sixth and the seventh blocks were used to evaluate real-life functioning. Taking into account the types of activity that were possible in residential psycho-neurological care facilities, we evaluated the following components of real-life functioning: physical self-care, household activity, labor activity, attending interest and study groups and cultural events, managing time, recreational activities and social interactions. The assessed variables included the formation and retention of skills, motivation for this type of activity, and the amount of assistance needed. So, when assessing physical self-care and household activity, with the framework of the facility regulations the following parameters were used: take initiative in physical self-care; performs independently, without assistance; needs regular organizing assistance to perform; needs active help; the skill is not formed; the skill is lost; not interested in performing. When assessing labor activity the following parameters were used: officially employed; motivated to work, carry out labor assignments regularly; declared willingness to work, not involved in labor assignments; not interested in labor, not involved in labor assignments. When assessing attending interest and study groups and cultural events the recorded parameters were: attend study-groups and cultural events with pleasure and on their own; needs regular organizing assistance to attend study-groups and cultural events; not interested in attending study groups or cultural events; does not visit; actively involved. When assessing social contacts the recorded parameters were: maintain close relationships (friendship or partnership) with residents; have acquaintances and people to interact with inside residential facility; have acquaintances and people to interact with outside residential facility; close relations with relatives; formal relations with relatives; relations with relatives are lost.

The form of the Protocol for each item related to real-life functioning included both a binary score (yes −1 or no – 0) for each of the recorded variables and a comment with a detailed meaningful description of the parameter, i.e., how exactly the subject implements this task in his real life. As a result we received a binary matrix for 100 samples that was analyzed.

Examination of 21 subjects using the BNSS scale was a pilot study of the applicability of this scale in residential care facilities in order to study the structure of negative disorders and identify targets for rehabilitation effects.

### 2.6. Statistical analysis

The quantitative results of the binary matrix were processed using Statistica 13.0 software.

When describing the demographic and social characteristics of the subjects of various groups, statistical methods were used: descriptive statistics (mean and standard deviation), frequency analysis. Comparability of groups by age and duration of living in a residential psycho-neurological care facility was assessed by Student’s *T*-test.

To compare the frequency of each of the parameters related to real-life functioning between the groups we used Chi-square test. The Yates correction was applied when an attribute’s frequency was <5.

We compared females with schizophrenia with males with schizophrenia to address the main objective of the study.

To analyze the results of BNSS in the pilot part of the study, we used Mann–Whitney *U*-Test and the method of K-means clustering, which from a computational point of view opposite to an analysis of variance. K-means cluster analysis allows to a priori select a researcher-specified number of clusters to divide the subjects into groups in such a way that the differences in the estimated parameters between the subjects of one cluster are minimal, and the variability between different clusters is maximum. The number of clusters is specified either on the basis of the hypothesis that the subjects belong to a certain number of distinct categories. In this case, cluster analysis allows to test the hypothesis (27). Or, as in the present study, the number of clusters is selected by recursive analysis in such a way that the explanation of the inclusion of specific subjects in specific groups does not intuitively contradict reality. In this case, a recursive formation of a hypothesis takes place, which must be tested in the future.

## 3. Results

The results obtained during the examination of the patients were analyzed taking into account the concept of Real-life functioning (15, 16). In accordance with it, self-care and housekeeping skills are necessary for independent living, work orientation, independent organization of leisure is associated with productivity, and interpersonal communication is associated with social interaction. Thus, the following “Functional outcomes” were studied in the examined patients: self-service, housekeeping skills, orientation to work and labor activity, attending vocational activities and entertainment events, independent organization of leisure, interpersonal relations.

### 3.1. Physical self-care

In residential facilities, physical self-care or personal hygiene could be maintained in accordance with the facilities’ regulations. Thus, bath days were usually held twice weekly, bed linen was changed once in two weeks, and hair-dressing services were provided once a month. In the matters of personal hygiene, however, the inmates were free to act on their own initiative. The inmates could take a shower on their own at any time, had they so desired, choose how often to send their washing to the laundry, wash by hand the items that require delicate washing, find additional ways to have their nails and hair done regularly. Such initiatives in their self-care were taken by 10.9% of females with schizophrenia but not by male subjects. Fifty two percent of females with schizophrenia, 51.9% of males with schizophrenia maintained their personal hygiene on their own with the framework of the facility regulations. Among those who needed regular organizing assistance (such as reminding patients to take a shower, change bed linen and send their washing to the laundry) were 26% of females with schizophrenia and 35% of males with schizophrenia. More active help with their physical self-care was needed by 10.9% of females with schizophrenia, 13% of males with schizophrenia. The results of comparing the groups, using a chi-square test (Table 2), showed that the groups differed significantly in only one parameter: females with schizophrenia, living in residential facilities, were taking extra initiative in their physical self-care significantly more often than men.

**Table 2:** Physical safe-care.

	Females with schizophrenia (*N* = 46)	Males with schizophrenia (*N* = 54)	Chi-square/Yates corrected Chi-square	*p*
Take initiative in physical self-care	5	0	6.18/4.10	0.013*/0.0428
Self-care without assistance with the framework of the facility regulations	24	28	0.00	0.97
Needs regular organising assistance with physical self-care with the framework of the facility regulations	12	19	0.96	0.327
Needs active help with physical self-care with the framework of the facility regulations	5	7	0.10	0.748

*Differences are significant.

### 3.2. Household activity

The possibilities for household activities were also limited by the facility regulations. The possibilities for the inmates to cook their own meals were only provided in one residential facility. Only 4.3% of females with schizophrenia and 5.6% of males with schizophrenia lived in apartment-type rooms where inmates were expected to clean up by themselves. None of the facilities provided any opportunities for the inmates to use a washing machine or a vacuum cleaner by themselves. Nevertheless, 13 of females with schizophrenia (28.2%), 9 males with schizophrenia (16.6%) (Chi-square = 1.95, *p* = 0.16) worked toward refurbishing their rooms (purchased furniture items and home appliances), preferred to do the home chores by themselves (tidy up, clean, wash some clothing), and took maximum advantage of the possibilities available to them at the facility (used laundry services; where cooking facilities were available, bought groceries and kitchenware and cooked meals). At the same time, where both females and males with schizophrenia did not engage in household activities, it seems in accordance with our observations and explanations of the subjects that it was associated more with their lack of interest and insufficient motivation. Both females and males with schizophrenia who did not engage in any household activities mentioned that, at the facility, “the staff do the cleaning,” “the food is good here,” or referred to the lack of necessary appliances. Some mentioned the prohibitions for the inmates to tidy up their rooms or cook by themselves, which did not exist in reality. For one female with schizophrenia doing no household activities was associated with immaturity or lack of relevant skills.

### 3.3. Work-mindedness and labor activity

Among the females with schizophrenia, there were significantly fewer of those not interested in any kind of labor and did not do any vocational assignments than among the males with schizophrenia. The females with schizophrenia were work-minded, motivated to work and did vocational assignments significantly more often than males with schizophrenia. Such assignments were typically associated with cleaning the grounds or helping at the cafeteria. Both female and male subjects with schizophrenia, who refused to do assignments offered to them and declared their willingness to work, were, as a rule, minded toward highly skilled labor in line with their profession acquired earlier. They talked about their desire to get a job as a teacher of foreign language, interpreter, or journalist. At the same time, the desires they voiced often did not match their abilities and could not be fully fulfilled as employment. The results of comparing the groups, using a chi-square test, are presented in Table 3.

**Table 3:** Labor activity.

	Females with schizophrenia (*N* = 46)	Males with schizophrenia (*N* = 54)	Chi-square/Yates corrected Chi-square	*p*
Employed in residential facility	3	2	0.42/0.03	0.51/0.85
Motivated to work, carry out labor assignments regularly	18	11	4.25*	0.039
Declared willingness to work, not involved in labor assignments	8	10	0.02	0.88
Not interested in labor, not involved in labor assignments	17	31	4.16*	0.04

*Differences are significant.

### 3.4. Attending interest and study groups and cultural events

Compared to the males with schizophrenia, the females with schizophrenia showed greater interest in hobby and study groups, classes, and cultural events, held at the facilities as well as in offsite excursions. The females more often attended these on their own while males needed organizing assistance in a form of personal invitations and additional incentives. The number of females with schizophrenia who refused to participate despite the facility staff’s attempts to involve them in such activities did not differ significantly from that of males. The results of comparing the groups, are presented in Table 4.

**Table 4:** Attending interest and study-groups and cultural events.

	Females with schizophrenia (*N* = 46)	Males with schizophrenia (*N* = 54)	Chi-square/Yates corrected Chi-square	*p*
Attend study-groups and cultural events with pleasure and on their own	20	9	8.67*	0.032
Needs regular organizing assistance to attend study-groups and cultural events	15	31	6.15*	0.013
Not interested in attending study groups or cultural events, not involved	11	14	0.05	0.817

*Differences are significant.

### 3.5. Subjects’ initiative in managing their time including recreational activities

Twenty females with schizophrenia, 10 males with schizophrenia (Chi-square = 7.37*, *p* = 0.0066) reported that they managed their day by themselves. They were able to name their routine activities during the day, their hobbies, etc.

The females with schizophrenia who managed their day by themselves regularly ran errands associated with cleaning the grounds and helping in the kitchen and cafeteria. They participated in the preparations for cultural events, sat on community boards of the users of social services, read books, spend time with female friends (usually very few), attended interest groups, embroidered, drew, kept journals, took notes of their reading so as to better memorize it or wrote short stories.

The males with schizophrenia who managed their day themselves also often reported about running errands (doing vocational assignments) and, less frequently, about creative activities, being more inclined to watch news channels. They were more selective in their choice of study and interest groups. Many of them reported subjectively experienced inactivity: “I watch the telly, read a book, thumb through a magazine, make tea. On the whole, do nothing, dust the room every day.”

### 3.6. Interpersonal relationships

Friendly relationships with other inmates were maintained by 36.9% of females with schizophrenia and 20.37% of males with schizophrenia. Females with schizophrenia usually had one or two female friends they regularly spent time with, drank tea, discussed personal matters, shared news and plans, and watched TV (entertainment and news programs). Males with schizophrenia who regularly interacted with other inmates shared personal information less often than females and tended to discuss politics and sports more often.

For females and males with schizophrenia, the circle of their contacts outside their residential facility consisted of persons they knew before their placement. The results are presented in Table 5.

**Table 5:** Interpersonal relationships.

	Females with schizophrenia (*N* = 46)	Males with schizophrenia (*N* = 54)	Chi-square	*p*
Maintain close relationships (friendship or partnership) with residents	17	11	3.39	0.066
Have someone to communicate and interact with inside residential facility	21	14	4.25*	0.039
Have someone to communicate and interact with outside residential facility	10	8	0.81	0.369
Close relations with relatives are not lost	10	8	0.81	0.369
Relations with relatives are formal	22	30	0.59	0.44
Relations with relatives are lost	14	16	0.01	0.93

*Differences are significant.

### 3.7. Negative symptoms and real-life functioning in patients with schizophrenia

**Figure 1:**
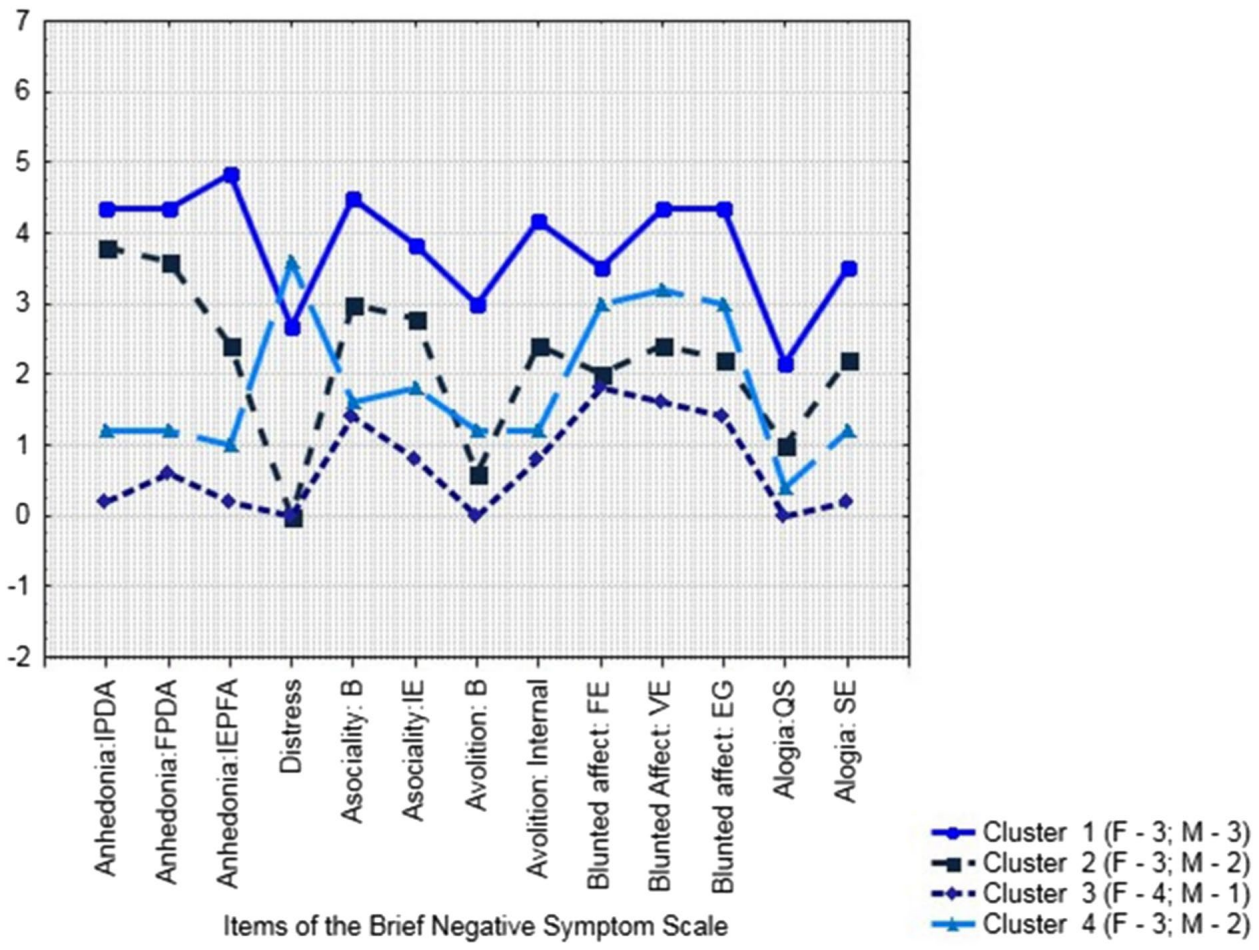
Cluster analysis of BNSS results (*N* = 21: Females–13; Males–8): Plot of Means.

The results of the pilot evaluation of BNSS negative symptoms in a part of subjects with schizophrenia (*N* = 21) are presented in Table 6. Comparing BNSS total score and subscale scores in females and males with schizophrenia with Mann Whitney *U* test did not define significant differences. Then, with K-means cluster analysis we divided the subjects into 4 clusters (Figure 1; Table 7).

**Table 6:** BNSS scores in females and males.

	Females with schizophrenia (*N* = 13) Mean ± SD; Min – Max	Males with schizophrenia (*N* = 8) Mean ± SD; Min – Max	*U*	*p*-value
BNSS total score	28.77 ± 17.2; 6–61	28.50 ± 15.41; 7–49	50.5	0.94
Anhedonia: Intensity of pleasure during activities	2.46 ± 2.03; 0–5	2.50 ± 2.00; 0–5	51.0	0.97
Anhedonia: Frequency of pleasure during activities	2.54 ± 1.90; 0–5	2.50 ± 2.07; 0–5	51.5	1.00
Anhedonia: Intensity of expected pleasure from future activities	2.46 ± 2.47; 0–6	1.88 ± 1.96; 0–5	45.5	0.66
Distress	1.31 ± 1.80; 0–5	2.13 ± 1.89; 0–4	38.0	0.33
Asociality: behavior	2.85 ± 2.03; 0–6	2.50 ± 1.41; 1–5	46.0	0.69
Asociality: internal experience	2.31 ± 2.02; 0–5	2.50 ± 1.85; 0–5	47.5	0.77
Avolition: behavior	1.31 ± 1.38; 0–3	1.25 ± 1.75; 0–4	50.0	0.91
Avolition: internal experience	2.15 ± 1.68; 0–5	2.38 ± 1.92; 0–5	48.0	0.80
Blunted affect: Facial expression	2.77 ± 1.17; 1–5	2.38 ± 0.92; 1–4	43.0	0.54
Blunted affect: Vocal expression	3.15 ± 1.68; 0–6	2.63 ± 1.19; 1–4	39.0	0.37
Blunted affect: Expressive gestures	3.00 ± 1.63; 0–5	2.50 ± 1.51; 0–4	42.0	0.49
Alogia: Quantity of speech	0.85 ± 1.46; 0–5	1.13 ± 1.13; 0–3	40.0	0.40
Alogia: Spontaneous elaboration	1.69 ± 2.14; 0–5	2.13 ± 1.81	43.0	0.54

**Table 7:** Cluster analysis of BNSS results.

	Cluster 1 *N* = 6 (M ± SD; Var)	Cluster 2 *N* = 5 (M ± SD; Var)	Cluster 3 *N* = 5 (M ± SD; Var)	Cluster 4 *N* = 5 (M ± SD; Var)
Females/Males	3/3	3/2	4/1	3/2
Anhedonia: Intensity of pleasure during activities	4.3 ± 0.5; 0.27	3.8 ± 1.1; 1.2	0.2 ± 0.4; 0.2	1.2 ± 1.3; 1.7
Anhedonia: Frequency of pleasure during activities	4.3 ± 0.5; 0.27	3.6 ± 1.1; 1.3	0.6 ± 0.9; 0.80	1.2 ± 1.6; 2.7
Anhedonia: Intensity of expected pleasure from future activities	4.8 ± 1.2; 1.37	2.4 ± 2.3; 5.3	0.2 ± 0.4; 0.2	1.0 ± 1.0; 1.0
Distress	2.7 ± 1.5; 2.27	0.0 ± 0.0; 0.0	0.0 ± 0.0; 0.0	3.6 ± 0.9; 0.8
Asociality: behavior	4.5 ± 1.2; 1.50	3.0 ± 0.7; 0.50	1.4 ± 1.7; 2.80	1.6 ± 1.5; 2.30
Asociality: internal experience	3.8 ± 1.6; 2.57	2.8 ± 2.0; 4.2	0.8 ± 1.1; 1.20	1.8 ± 1.6; 2.70
Avolition: behavior	0.6 ± 0.6; 0.40	0.6 ± 0.9; 0.80	0 ± 0; 0	1.2 ± 1.6; 2.7
Avolition: internal experience	4.2 ± 0.7; 0.57	2.4 ± 1.5; 2.30	0.8 ± 0.8; 0.7	1.2 ± 1.3; 1.7
Blunted affect: Facial expression	3.5 ± 1.0; 1.10	2.0 ± 0.0; 0.0	1.8 ± 0.8; 0.7	3.0 ± 1.0; 1.0
Blunted affect: Vocal expression	4.3 ± 0.8; 0.67	2.4 ± 0.5; 0.30	1.6 ± 1.8; 3.30	3.2 ± 1.1; 1.2
Blunted affect: Expressive gestures	4.3 ± 0.5; 0.27	2.2 ± 1.6; 2.7	1.4 ± 0.9; 0.8	3.0 ± 1.4; 2.0
Alogia: Quantity of speech	2.1 ± 1.5; 2.17	1.0 ± 1.4; 2.0	0 ± 0; 0	0.4 ± 0.5; 0.30
Alogia: Spontaneous elaboration	3.5 ± 1.6; 2.70	2.2 ± 2.1; 4.70	0.2 ± 0.4; 0.20	1.2 ± 1.8; 3.20

The first cluster comprised the subjects whose condition, assessed with the Brief Negative Symptom Scale (BNSS), was characterized by predominant anhedonia/asociality/avolition complexes. In a situation of residential psycho-neurological care facility, both females and males avoided contacts with other people and were inert, passively complying with the regulations. The subjects from this group needed help with their personal care and did not get involved in any rehabilitation activities.

In the subjects who comprised the second cluster, negative symptoms were less pronounced with the same deficit structure. By the time of this survey, four subjects had productive symptoms such as delusional ideas of reference, persecution, or importance, which largely determined their interaction with other people and everyday functioning. The patients were active in their contacts with other people, pushing their ideas.

The fourth cluster also included the subjects with schizophrenia whose need in residential care was dictated by their productive symptoms that persisted for many years. Negative symptoms were less pronounced in this group than in the first cluster and comparable to the second cluster. Negative symptoms profile, however, differed from the rest of the clusters, with distress deficit being the most pronounced. In other words, the subjects with schizophrenia in this group were not distressed either about the symptoms of their illness or their social outcomes, which in the end brought them into residential care facilities.

What interested us most was the third cluster that comprised 4 female and 1 male subjects. These patients did not have any productive symptoms for years (from 3 years in the youngest female subject to 15 years), their condition was evaluated by psychiatrists to be sustained remission of psychiatric disorder; their negative symptoms were the least pronounced, with the predominant complex of reduced expressiveness and internal experiences, while their level of real-life functioning was the highest. They were active and independent in their physical self-care, managed their household activities by themselves, engaged in active pastime including hobbies, self-learning, and social activities, and were minded toward socially beneficial activities or employment (the younger subjects). The subjects who comprised this cluster were active in their social contacts, had close friendships with one or two persons and a wider circle of contacts, and were active in maintaining or restoring their relationships with their families. The relevant professionals at their residential facilities regarded them eligible for nonresidential care.

### 3.8. Discussion

Our observational study has found certain differences between men and women, chronically ill with schizophrenia and living in residential psycho-neurological care facilities. More female subjects with schizophrenia remained active and took initiative in their physical care, vocational activities, and involvement in cultural events than male subjects. Females with schizophrenia – more often than males – had steady circles of companions at their residential facility and maintained friendly relationships with other inmates, including sharing personal information with them.

This observation is in line with the concept of decreased social inclusion of patients with schizophrenia. Thus, according to Killaspy et al. (28), the main domains of social inclusion (productivity and social integration) change over the time since the onset of a psychotic illness, with patients becoming less productive and less socially integrated. The more productive and the better socially functioning the patients were before the onset of their psychosis, the more profoundly their illness affected their social integration and productivity. It may be assumed that persons who have been better socially integrated prior to being placed in residential care, when they find themselves in a facility for persons with severe chronic psychiatric disorders, are more susceptible to adverse impact of its environment.

The observed differences in everyday functioning between females and males with schizophrenia, on the one hand, could be explained by the earlier onset of schizophrenia in males, as according to the neurodevelopmental theory of schizophrenia, with an earlier onset of symptoms, including the prodromal phase, there is a slowing down or arrest of the development of social cognitions. On the other hand, according to Abel K. et al. (29), men have difficulties with social adaptation even during the premorbid period more often, and negative and depressive symptoms may be more pronounced in men than in women.

These findings can also be partially explained by the aforementioned estrogen hypothesis. It was shown that both males and females with schizophenia have lower estrogen blood levels than the healthy population. While females have higher 17β-Estradiol concentration in the brain, this performs as both a neuroplasticity increasing factor and anti-apoptotic factor through different ways of activity with receptors and mitochondria of neurons. Another meaningful factor is related to a higher stress resilience in females through hypothalamic–pituitary–adrenal pathway, as it was shown in several recent studies (30–32).

While estrogen levels reduce during menopause, this can potentially be attributed as a meaningful factor to observed differences in female population of the residential care facilities given the mean ages in the groups. In the current study this can only be speculated as no lab tests were performed during the study. However this might be another potentially significant direction for future research, especially in the light of individualized approach to antipsychotic and augmentation treatments (18, 33, 34).

On the whole, patients with schizophrenia can demonstrate poorer everyday functioning in residential facilities due to impaired motivation, which is characteristic of this illness. The fact that the staff of residential social care facilities lacks necessary skills for working with such impairments leads to this group of inmates, with lack of external motivation, being left to their own devices and excluded from rehabilitation processes.

Our study has also revealed the deficit of rehabilitation measures in residential social care facilities. Such measures usually aim at promoting the formation of elementary household and social skills in the patients and organizing their leisure time, and neither address each patient’s individual problems nor take into account particular features of social and cognitive deficit in schizophrenia. The content of measures aimed at organizing patients’ leisure time typically does not meet the expectations and needs of patients with schizophrenia and thus fails to stir their interest.

Placement of patients with schizophrenia in residential facilities should be associated with a more severe or longer course of the illness and impaired ability to live an independent, autonomous life. In other words, only those patients with schizophrenia should be placed in such institutions, for whom nonresidential forms or care are ineffective. We have, however, found that the sample of patients with schizophrenia in our study was heterogeneous. K-means cluster analysis of the BNSS scores made it possible to presumably distinguish 4 groups with different profiles of negative symptoms, in which daily functioning also differed. We have potentially identified a group of patients in sustained remission, with less pronounced negative symptoms and a higher level of social functioning. It was probably due to scarcity of alternative forms of community care and, in these situations, placement in residential facilities has been the only available form of social support. These preliminary results allow planning future studies.

It should be mentioned that residential social care in the institutions for psychiatric patients is often an additional factor that adversely affects their real-life functioning due to both the limitations of available forms of productive activity and the manifestations of hospitalism and aggravated stigmatization and self-stigmatization.

While institutions were once seen as the best way of caring for people with severe mental disorders, provision of free, healthy and supportive environment in the community to promote recovery is presently regarded as one of the main aspects of effective care for patients with schizophrenia (3). The process of deinstitutionalization has been going on in many European countries over the last decades (35). At-home or community care, which is beneficial for inclusion of psychiatric patients with disability, reduction of stigma, and realization of these people’s rights to a free and active life, involvement in the life of society, employment, and founding a family, is seen as a high priority alternative to institutional forms of care in residential facilities. At the same time, residential social care facilities for persons with mental disorders still exist in many European countries. According to the latest published report on deinstitutionalization of social care facilities, 1.5 million persons still live in residential facilities in 27 EU states (36). In Russia where the reform of social care system for people with mental disorders only began to be implemented in the last few years, the residential form of social care provision to persons with chronic mental conditions is still widespread. According to the national full-sized non-control observational descriptive study conducted in Russia in 2019, more than 150,000 adults live in residential social care facilities, intended for persons with mental disorders (37). According the oral report of Kekelidze Z. (2020) on the results of the study, patients with schizophrenia comprise 46.9% of all persons living in residential psycho-neurological care facilities. At the same time, while, in the 18–30 and > 70 age groups, most of the persons placed in such facilities are individuals with intellectual disabilities and dementia, respectively, the main cause of placement in residential facilities among the 30–60 age group is severe schizophrenia with poor functional outcomes. The comparison of the number of men and women, placed in residential facilities, showed that there were significantly more men then women in all age groups.

It appears that our finding of a greater percentage of women than men among the patients who performed well in real life was associated with the shortcomings of primary psychiatric care as well as with poorly organized system of community social care rather than with gender differences in the outcomes in schizophrenia. There is a problem of underdiagnosing schizoaffective disorders, which are more common in women, and of not always justified change of diagnosis from episodic schizophrenia with a stable deficit to continuous schizophrenia. In Russia, there is no efficient system for promoting maximum possible working ability of patients with schizophrenia and for preventing their exclusion from their pre-existing circle of social relationships due to the lack of coherent interdepartmental interaction between psychiatric (mental health) and social care services. In this situation, growing social isolation and hospitalism lead to patients’ disablement and placement in residential social care facilities even when not every possibility for their living outside such institutions has been exhausted.

### 3.9. Study limitations

The subjects’ pre-established diagnoses have not been revisited, psychometric methods that could allow comparing intensity of productive symptoms have not been used, and severity of BNSS negative symptoms were only assessed in a part of the patient group.

We do not discuss pharmacological treatment here, hence this is another very important factor of the problem of residential facilities.

## Data availability statement

The data analyzed in this study is subject to the following licenses/restrictions: Institutional Requirements. Requests to access these datasets should be directed to OR, rusakovskaya.o@serbsky.ru.

## Ethics statement

The studies involving human participants were reviewed and approved by V. Serbsky National Medical Research Centre for Psychiatry and Narcology. The patients/participants provided their written informed consent to participate in this study.

## Author contributions

OR and OP contributed to conception and design of the study. OR collected the material, organized the database, performed the statistical analysis, analyzed the results, and wrote sections of the manuscript. OP and LM revised the first draft, analyzed the results, and wrote sections of the manuscript. OP wrote a literature review especially. NK revised the results and the manuscript. All authors contributed to manuscript revision, read, and approved the submitted version.

## Conflict of interest

The authors declare that the research was conducted in the absence of any commercial or financial relationships that could be construed as a potential conflict of interest.

## Publisher’s note

All claims expressed in this article are solely those of the authors and do not necessarily represent those of their affiliated organizations, or those of the publisher, the editors and the reviewers. Any product that may be evaluated in this article, or claim that may be made by its manufacturer, is not guaranteed or endorsed by the publisher.
